# The Correlation between Two Angiotensin-Converting Enzyme Inhibitor’s Concentrations and Cognition

**DOI:** 10.3390/ijerph192114375

**Published:** 2022-11-03

**Authors:** Attila Nagy, Réka Májer, Enikő Csikai, Adrienn Dobos, Gábor Süvegh, László Csiba

**Affiliations:** 1 Department of Health Informatics, Faculty of Health Sciences, University of Debrecen, 4032 Debrecen, Hungary; 2 Department of Neurology, Faculty of Medicine, University of Debrecen, 4032 Debrecen, Hungary; 3 Doctoral School of Health Sciences, Institute of Behavioral Science, Faculty of General Medicine, University of Debrecen, 4032 Debrecen, Hungary; 4 Department of Forensic Toxicology, Hungarian Institute for Forensic Sciences, 1903 Budapest, Hungary

**Keywords:** ACE inhibitors, lisinopril, enalapril, cognitive tests

## Abstract

Both lisinopril and enalapril are angiotensin-converting enzyme (ACE) drugs and widely used in the treatment of hypertension. Enalapril does not cross the blood–brain barrier, but lisinopril is centrally active. Our goal was to find out if there was a link between the actual concentration of ACE inhibitors and cognition and if there was a detectable difference between the two types of ACE inhibitors. Asymptomatic, non-treated patients were diagnosed by screening and the hypertension was confirmed by ambulatory blood pressure monitoring (ABPM). A battery of cognitive tests was used to assess the impact of randomly assigning participants to receive either lisinopril or enalapril. All neurocognitive functions were measured, especially the most affected by conditions of compromised perfusion pressures, such as hypertension, which are attention and executive functions. The lisinopril concentration showed a significant inverse correlation with mosaic test (coeff. = −0.5779) and seemed to have a significant negative effect on perceptual motor skills (coeff. = −0.5779), complex attention (coeff. = −0.5104) and learning (coeff. = −0.5202). Compared with enalapril, lisinopril is less successful in improving the components of cognitive functions.

## 1. Introduction

Hypertension increases the risk of cardiac issues, renal diseases and stroke. It affects about 1.28 billion people aged 30–79 around the world, with two-thirds of them living in low- and middle-income countries [1].

Alzheimer’s disease and vascular dementia are the most common types of dementia and both are linked to chronic arterial hypertension, which is a well-known risk factor [2].

The ACE plays a crucial role in the regulation of blood pressure. ACE inhibition has a general antihypertensive effect [3].

ACE is produced by neurons and converts angiotensin I to angiotensin II [4,5]. In a mouse model of Alzheimer’s disease, ACE inhibitors have been shown to reduce microglial activation and neuronal damage [6]. ACE inhibition has also been shown to improve cognitive function, especially memory, in Alzheimer’s patients in a number of clinical trials and observational studies [7,8,9,10,11,12,13]. Thus, ACE inhibition is a strong candidate in clinical trials to alleviate cognitive dysfunction [14].

Lisinopril has a few key properties that differ from enalapril; (1) it has a long half-life, (2) it is hydrophilic, and (3) it is not broken down by the liver and crosses the blood–brain barrier. Lisinopril is a competitive inhibitor of ACE. It stops angiotensin I from changing into the powerful vasoconstrictor angiotensin II. Its pharmacological properties make it suitable for the treatment of hypertension [15,16].

Enalapril is also an ACE inhibitor, which is also widely used in the treatment of hypertension, with a similar pathomechanism to lisinopril, however, enalapril does not cross the blood–brain barrier.

Our goal was to find out if there was a link between the actual concentration of ACE inhibitors and cognition in relatively young hypertensives without cognitive deficit and if there was a detectable difference between the two types of ACE inhibitors.

## 2. Materials and Methods

Our research was performed at the Neurological Department of the Clinical Centre of the University of Debrecen. Neurologically asymptomatic non-treated hypertensive patients were found by screening of policemen and firemen of the city. None of them had structural abnormalities on the cranial CTs. The hypertension was confirmed by ABPM and the effects of randomly assigned lisinopril or enalapril were investigated with the battery of cognitive tests before and after 3-month antihypertensive therapy. A battery of cognitive tests were applied. The actual blood concentration of enalapril and lisinopril concentrations were measured, and the cognitive functions were also measured simultaneously.

### 2.1. Statistical Analysis

Proportions, means and medians were used for descriptive statistics. The Shapiro–Wilk test evaluates normality for continuous variables. Student’s *t*-test was used to compare averages of baseline and 3-month parameters. For quantification of associations between continuous variables, Pearson’s correlations were applied.

Statistical analyses were performed by Intercooled Stata v13 [17].

### 2.2. Ethics Approval

The study protocol was approved by the Ethics Committee of the University of Debrecen, and the study was performed in accordance with the Declaration of Helsinki (registration number: 4700168520, file number: KK/186/2016). After reading the protocol, all the participants gave their written permission to take part.

### 2.3. Procedure

All patients underwent a detailed 90 min long neuropsychological evaluation with the guidance of a psychologist. The test was designed to determine the main neurocognitive functions listed in the 5th Edition of the Diagnostic and Statistical Manual of Mental Disorders (DSM-5): reaction time, attention, executive function, learning, memory and perceptual motor skills. The test package had the most sensitive tests that could pick up on small differences in cognitive function that might not be obvious in everyday life.

The order of tasks was changed by some subjects to control the effect of the order on performance (e.g., due to fatigue). Two key aspects were considered when the sequence of tasks was created. Exercises requiring creativity (e.g., five-point tests and verbal fluency tests) were performed earlier than the types of tasks that measured cognition. During the 20 min delay of the delayed-recognition task, only nonverbal tasks were performed to avoid interference. Prior to the baseline assessment, we drew randomly from these tests. All neurocognitive functions were measured, especially the most sensitive ones to hypertension, which are attention, executive functions, and memory.

The following tests were used to assess cognitive functions: computerized reaction time assessment, digit symbol substitution test [18,19,20], Toulouse–Piéron test [21,22], Victoria Stroop test [23,24], Trail Making Test [25], Five-Point Test [26,27,28], Digit Span Test [29,30], Corsi block-tapping test [30,31], Rey-AVLT [32,33,34], verbal fluency test: letter, category, and action fluency [35,36,37], WAIS mosaic test [18,19,20].

### 2.4. Laboratory Measurement

Sample preparation and extraction procedure were done according to Padua et al. (2004) with some modifications [38].

Sample preparation: 400 μL of aqueous hydrochloric acid solution (10 mM) containing 10 ng/mL trandolapril as an internal standard were dispensed in appropriate Eppendorf tubes and then 500 μL sample human blood was added and vortex-mixed. Waters HLB Oasis solid phase extraction (SPE) cartridges were preconditioned by washing first with 2 mL methanol and then 1 mL of hydrochloric acid solution (10 mM) under light vacuum. All blood samples were applied to the individual Waters Oasis SPE cartridges. Using light vacuum, the samples were slowly drawn through the cartridges. The cartridges were washed five times with 1 mL of aqueous hydrochloric acid solution (10 mM) under light vacuum. The cartridges were then eluted in separate glass tubes with 0.5 mL methanol by applying a light positive pressure. The solvent was evaporated by using a flow of nitrogen at room temperature. The dry residues were reconstituted with 200 μL of acetonitrile/water (30:70 *v*/*v*) and transferred to vials.

**Figure 1:**
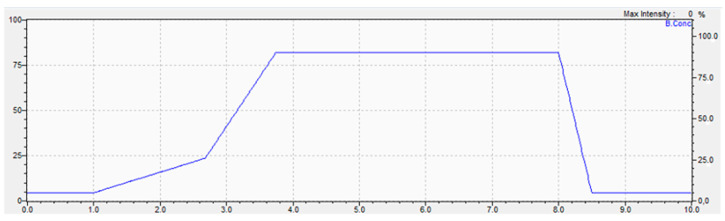
Chromatographic conditions.

Chromatographic conditions: An aliquot (10 μL) of each blood extract was injected into Phenomenex Kinetex C18 analytical column (100 mm × 3.0 mm × 2.6 μm), operating at 40 °C temperature in a Shimadzu Nexera X2 ultra high performance liquid chromatograph (UHPLC). The mobile phases were formiate buffer 15 mM pH 4.0 (A) and acetonitrile with 0.1% formic acid (B). The gradient begins with 5% B eluent through 1 min, then increases to 26% B eluent for 2.68 min, then rises to 90% B eluent at 3.74 min. It remains isocratic until the 8th minute, then decreases back to 5% B eluent through 0.5 min and holds it for 1.5 min (Figure 1).

The mass spectrometer was a Shimadzu LCMS 8050 equipped with an electrospray ion source used in positive mode and in multiple reaction monitoring (MRM) mode. The monitored MRM transitions can be seen in Table 1.

The calibration points were 6.25, 12.5, 25, and 40 ng/mL for enalapril and lisinopril and 12.5, 25, 50, and 80 ng/mL for enalaprilat. Limit of detection (LoD) values were under 1 ng/mL for every compound.

## 3. Results

The sample consisted of 34 patients, of whom 29.41% (10) were female. The average age ± SD was 49.53 ± 11.14 years. Enalapril was administered to 18 patients (52.94%), while lisinopril was the used medication for 16 patients (47.06%).

Lisinopril (contrary to enalapril) not only significantly decreased the diastolic but also the systolic blood pressure (146.36 mmHg vs. 137.86 mmHg *p* = 0.004) (Table 2).

The lisinopril concentration showed a significant inverse correlation with mosaic test (coeff. = −0.5779).

Lisinopril (contrary to enalapril) seemed to have a significant negative effect on perceptual motor skills (coeff. = −0.5779), complex attention (coeff. = −0.5104), and learning (coeff. = −0.5202) (Table 3).

## 4. Discussion

In our pilot study, we measured the concentrations of enalapril and lisinopril and compared them with the results of cognitive tests after 3 months therapy. In our pilot study, the lisinopril worsened some important elements of cognition (e.g., attention, visuospatial orientation). This deleterious effect was concentration-dependent and not related to the blood pressure decrease. Neither the systolic nor the diastolic values showed significant differences between the enalapril and lisinopril group after therapy; therefore, the negative impact of lisinopril on the cognition could be mediated by its direct effect on CNS. Others also detected a negative effect of lisinopril therapy. Hajjar et al. found an increased number of white matter abnormalities in the lisinopril-treated patients after a relatively short therapy [7].

It is not yet determined whether the diuretics, calcium channel blockers, beta-blockers, angiotensin-converting enzyme inhibitors, and angiotensin receptor blockers (ARBs)) are associated with different effects on cognition. Some antihypertensive medications were linked to a decreased risk of dementia in elderly people with isolated systolic hypertension [39]. ARBs, particularly those that cross the BBB, are linked to better memory preservation and lower WMH volume than other antihypertensive drugs [40].

There is a large body of research evidence that ACE inhibitors also have beneficial effects on cognition in addition to regulating blood pressure [41].

The effect of centrally acting ACE inhibitors on cognition of hundreds of demented patients were most evident within the first 6 months of treatment [42].

Recent observations also showed that this effect can be separated from the effect of lowering blood pressure [16].

A 2014 study examined the effect of ACE inhibitors on memory performance in 205 healthy subjects. Their results showed that the ACE gene affects memory performance, and thus that the use of ACE inhibitors affects cognitive function [43].

However, the observations are controversial. A previous study has already shown that blood pressure regulators that penetrate the brain are associated with protection against cognitive decline [20] but others could not support the positive effect of centrally active ACE inhibitors on cognitive functions [16].

Our previous observation also confirmed the beneficial effect of 3 month ACE inhibitor therapy on cognition, but in that study a mixed group of ACE inhibitors (CNS-active and non-active) were investigated and we did not measure the drug concentrations in the blood [44].

The majority of previous studies investigated the long-term antihypertensive therapies either on prevention/slow downing of cognitive decline of mild cognitive impairment or demented patients.

Italian authors evaluated cognitively normal individuals treated for hypertension with a 3.5-year follow-up. They found that the enalapril alone or combined with the lisinopril were associated with a reduced risk of incident MCI [45].

In another study, 98 mild-to-medium severity hypertensive patients were treated for six weeks with different classes of antihypertensive drugs (including enalapril). The therapy was associated with some small decrements in psychomotor performance and small improvements in working memory [46].

Fogari et al. investigated patients with mild-to-moderate essential hypertension and treated with valsartan or enalapril for 16 weeks. The enalapril did not deteriorate the cognition but did not result in any improving effect. A similar result was published in a 2004 study comparing enalapril with another class of drugs, which found no significant change in cognitive function in the group of enalapril users on any of the cognitive function test scores [47].

In patients with mild cognitive impairment and hypertension, 1-year therapy with lisinopril resulted in significant accumulation/worsening of white matter lesions in the brain [48].

In several elements of emotional, cognitive, and social functioning, lisinopril outperformed metoprolol; however, it was inferior to telmisartan in other studies [40,48].

Our study differed from the observation cited above. We collected relatively young hypertensives without cognitive impairment.

As for our knowledge, our study is the first one that investigated not only the actual concentrations of enalapril and lisinopril in the blood but also the cognition at the same time.

One of the main questions was whether there is a detectable difference in cognitive function between a blood–brain barrier crossing (centrally active) ACE inhibitor (lisinopril) and a blood–brain barrier (non-crossing) ACE inhibitor (enalapril) [18,49,50]. A 2009 study involving more than 1000 patients concluded a finding that after pooling the results of cognitive function tests, an advantage of blood–brain barrier crossing ACE inhibitors was seen [49].

Our study highlights differences related to cognitive functions by different drugs [47]. Compared with enalapril, lisinopril seemed to be less successful in improving the components of cognitive function.

Based on the concentration results, we do not see an advantage of centrally active ACE inhibitors in improving cognitive function. A higher lisinopril concentration was associated with worse performance in several cognitive areas. A previous study also suggested a decrease in executive function among people taking lisinopril [50].

Our pilot study has the following strengths: essential hypertensive patients who had received no therapy before the study had no concomitant confounding disease, single drug therapy caused no symptoms of mild cognitive impairment or dementia, there was no structural brain diseases (e.g., silent brain infarct, brain atrophy), and there was a wide spectrum of cognitive tests measurements of the actual concentration of the drugs.

The limitations of our study include the small number of patients and the unbalanced gender distribution (more males than females). The follow-up was relatively short and the change was analyzed after 3 months. Different effects may have been demonstrated over a longer period.

According to our findings, lisinopril and enalapril do not appear to be effective in terms of cognitive performance. We were not able to show that the ACE inhibitor had a positive effect on cognitive functions when it got into the central nervous system. In fact, higher drug concentrations were linked to worse results in some areas of cognitive function.

In the future, another study with larger number of patients should confirm the results of this pilot study.

## 5. Conclusions

The therapy of hypertension is evolving. There is an unmet need for targeted and personalized types and doses of antihypertensive medications, improving patient adherence, and not worsening their cognitive status. As the blood pressure values did not differ significantly between the enalapril and lisinopril groups (before and after therapy); moreover, due to the negative effect of lisinopril on some elements of cognition, we assume that it is due to the direct effect of lisinopril on CNS. Larger prospective study should confirm these observations. If the larger studies confirm our results, those asymptomatic hypertensives who need sustained complex attention and visuospatial abilities in their daily work should avoid lisinopril therapy and choose another type of antihypertensive medication.

## Figures and Tables

**Table 1:** The monitored MRM transitions after automatic optimization.

Compound	Parention	Productions
Lisinopril	406.20	84.15; 246.25; 56.05
Enalapril	377.00	234.25; 117.15; 91.10
Enalaprilate (metabolite of Enalapril)	349.00	206.20; 117.15; 91.10
Trandolapril	431.20	234.25; 117.10; 130.30

**Table 2:** Baseline and 3-month characteristics of patients; bold indicates significant results of Student’s *t*-test.

Variable	Enalapril			Lisinopril		
	Before	After	*p*-Value	Before	After	*p*-Value
Simple reaction time	0.56 ± 0.11	0.52 ± 0.11	0.010	0.59 ± 0.08	0.57 ± 0.09	0.265
Selective reaction time	0.67 ± 0.13	0.65 ± 0.11	0.292	0.67 ± 0.06	0.68 ± 0.08	0.843
RAVLT total 1–5	48.83 ± 10.21	55.5 ± 8.66	0.001	50.2 ± 12.74	51.27 ± 10.46	0.598
RAVLT 6	10.78 ± 2.98	11.44 ± 2.75	0.210	10.33 ± 2.64	10.53 ± 3.42	0.677
Pieron	87.92 ± 12.81	91.68 ± 11.26	0.223	89.57 ± 11.31	91.97 ± 6.33	0.345
Trail Making	41.83 ± 17.62	41.61 ± 11	0.962	74.34 ± 58.53	54.77 ± 39.32	0.007
Digit span forward	6.22 ± 0.73	6.22 ± 1.17	1.000	6.13 ± 1.02	5.94 ± 1.18	0.549
Digit span backward	4.56 ± 0.51	5 ± 0.84	0.057	4.69 ± 1.08	4.56 ± 1.41	0.633
Digit symbol	41.15 ± 8.55	43.85 ± 10.78	0.064	38.95 ± 10.84	42.55 ± 8.07	0.056
Mosaic test	12.42 ± 2.35	12.83 ± 1.4	0.499	10.31 ± 4.03	11.77 ± 2.59	0.148
5 point test	95.35 ± 4.96	97.27 ± 5.33	0.043	93.72 ± 10.16	93.36 ± 11.25	0.892
Stroop test	23.77 ± 6.26	23.71 ± 5.7	0.951	27.76 ± 11.06	23.39 ± 10.13	0.015
Verbal fluency-letter	12.89 ± 4.89	15.56 ± 4.49	0.015	15.56 ± 4.44	17.56 ± 5.28	0.125
Verbal fluency-action	19.06 ± 5.07	19.29 ± 5.42	0.856	17.19 ± 7.02	18.19 ± 6.27	0.461
Verbal fluency-category	21.06 ± 4.44	20.29 ± 5.79	0.549	19.5 ± 5.77	19.13 ± 7.26	0.792
Corsi block forward	5.56 ± 1.04	5.83 ± 0.92	0.135	5.69 ± 1.14	5.56 ± 0.96	0.609
Corsi block backward	5.33 ± 1.41	5.5 ± 0.79	0.626	4.88 ± 1.09	5.5 ± 1.1	0.028
Letter fluency	12.33 ± 4.39	14.02 ± 5.11	0.257	13.75 ± 4.38	16.25 ± 4.39	0.015
Average fluency	16.39 ± 3.88	15.92 ± 5.08	0.698	16.33 ± 3.66	17.45 ± 4.17	0.029
Working memory	28.28 ± 2.3	29.22 ± 3.66	0.394	27.44 ± 4.53	27.69 ± 4.98	0.817
Language	98.29 ± 23.97	96.88 ± 30.81	0.853	98 ± 21.97	104.69 ± 25.01	0.029
Complex attention	−0.85 ± 2.1	−0.49 ± 2.39	0.272	−0.9 ± 2.16	−0.78 ± 2.05	0.718
Percepto-motor skills	12.42 ± 2.35	12.83 ± 1.4	0.499	10.31 ± 4.03	11.77 ± 2.59	0.148
Reaction time	−1.23 ± 0.23	−1.17 ± 0.2	0.063	−1.27 ± 0.11	−1.26 ± 0.14	0.659
Learning	62.61 ± 13.06	70.28 ± 10.72	0.006	64.27 ± 15.45	65.13 ± 13.04	0.750
ABPM_SBP	146.15 ± 11.96	140.31 ± 10.48	0.086	146.36 ± 5	137.86 ± 7.73	0.004
ABPM_DBP	85.92 ± 7.14	82.92 ± 5.3	0.047	90 ± 7.71	84.79 ± 7.23	0.016

**Table 3:** Correlation between cognitive field and drug blood concentration (Pearson’s correlation); only significant results are shown.

Cognitive Field	Drug	Pearson’s Coefficiens	*p*-Value
Perceptual motor functions	Lisinopril	−0.5779	0.039
Complex attention	Lisinopril	−0.5104	0.043
Learning	Lisinopril	−0.5202	0.047

## Data Availability

The data presented in this study are available on request from the corresponding author.
